# Understanding Whole-Person Health and Resilience During the COVID-19 Pandemic and Beyond: A Cross-sectional and Descriptive Correlation Study

**DOI:** 10.2196/38063

**Published:** 2022-05-16

**Authors:** Sripriya Rajamani, Robin Austin, Elena Geiger-Simpson, Ratchada Jantraporn, Suhyun Park, Karen A Monsen

**Affiliations:** 1 University of Minnesota Minneapolis, MN United States

**Keywords:** Omaha System, whole-person health, strengths, resilience, assessment, app, health information technology, health informatics, nursing, health care, mobile health, health application, mHealth, health data, health community, digital health

## Abstract

**Background:**

The COVID-19 pandemic has prompted an interest in whole-person health and emotional well-being. Informatics solutions through user-friendly tools such as mobile health apps offer immense value. Prior research developed a consumer-facing app MyStrengths + MyHealth using Simplified Omaha System Terms (SOST) to assess whole-person health. The MyStrengths + MyHealth app assesses strengths, challenges, and needs (SCN) for 42 concepts across four domains (My Living, My Mind and Networks, My Body, My Self-care; eg, *Income*, *Emotions*, *Pain*, and *Nutrition*, respectively). Given that emotional well-being was a predominant concern during the COVID-19 pandemic, we sought to understand whole-person health for participants with/without *Emotions* challenges.

**Objective:**

This study aims to use visualization techniques and data from attendees at a Midwest state fair to examine SCN overall and by groups with/without *Emotions* challenges, and to explore the resilience of participants.

**Methods:**

This cross-sectional and descriptive correlational study surveyed adult attendees at a 2021 Midwest state fair. Data were visualized using Excel and analyzed using descriptive and inferential statistics using SPSS.

**Results:**

The study participants (N=182) were primarily female (n=123, 67.6%), aged ≥45 years (n=112, 61.5%), White (n=154, 84.6%), and non-Hispanic (n=177, 97.3%). Compared to those without *Emotions* challenges, those with *Emotions* challenges were aged 18-44 (*P*<.001) years, more often female (*P*=.02), and not married (*P*=.01). Overall, participants had more strengths (mean 28.6, SD 10.5) than challenges (mean 12, SD 7.5) and needs (mean 4.2, SD 7.5). The most frequent needs were in *Emotions*, *Nutrition*, *Income*, *Sleeping*, and *Exercising*. Compared to those without *Emotions* challenges, those with *Emotions* challenges had fewer strengths (*P*<.001), more challenges (*P*<.001), and more needs (*P*<.001), along with fewer strengths for *Emotions* (*P*<.001) and for the cluster of health-related behaviors domain concepts, *Sleeping* (*P*=.002), *Nutrition* (*P*<.001), and *Exercising* (*P*<.001). Resilience was operationalized as correlations among strengths for SOST concepts and visualized for participants with/without an *Emotions* challenge. Those without *Emotions* challenges had more positive strengths correlations across multiple concepts/domains.

**Conclusions:**

This survey study explored a large community-generated data set to understand whole-person health and showed between-group differences in SCN and resilience for participants with/without *Emotions* challenges. It contributes to the literature regarding an app-aided and data-driven approach to whole-person health and resilience. This research demonstrates the power of health informatics and provides researchers with a data-driven methodology for additional studies to build evidence on whole-person health and resilience.

## Introduction

With more than 460 million cases of COVID-19 and more than 6 million deaths globally due to the pandemic as of March 2022 [1], along with the physical, financial, and emotional toll on the population, there is a critical need to renew focus on whole-person health and emotional well-being [2]. Whole-person health aims to help and empower individuals to improve their health in biological, behavioral, social, and environmental areas that are interconnected [3]. This whole-person health approach shifts the clinical and public health paradigms from limited transactional and disease-specific treatments to assessing and fostering overall health and promoting resilience [3].

This is known as strengths-based care, where the focus is on solutions and possibilities based on strengths (poststructuralist) models, with a shift away from deficit/pathology (structuralist) models that focus on problems and causes [4]. Strengths are defined as skills, capacities, actions, talents, potential, and gifts in each individual, family, and community [5]. The resilience of an individual is the ability to persevere, heal, and transform in the face of challenges, setbacks, and conflicts [6,7], and is dynamic across the life span [8], is applicable to mental health [9], and can be characterized using their strengths [7]. Movement toward a model that emphasizes talents and preferences is likely to benefit all persons, especially marginalized populations as the focus is on strengths [10], instead of deficits, and may help in decreasing stigmatization and improving engagement.

Informatics solutions through user-friendly tools such as apps for data collection and standards for data representation are useful for whole-person health assessments. A standardized terminology, Simplified Omaha System Terms (SOST), captures all of health in four domains: environmental, psychosocial, physiological, and health-related behaviors [11]. The Omaha System has been used as a strengths-based data capture model [12] and to operationalize resilience. It is mapped to clinical terminologies such as Systemized Nomenclature of Medicine–Clinical Terms (SNOMED CT) [13] and Logical Observation Identifiers Names and Codes, and is embedded within electronic health records (EHRs). In health care, strengths data should be considered in the context of problems so that the data is meaningful and adds value to improving health and health outcomes [14]. These tools with standardized data facilitate the integration of a consumer’s whole-person strengths, challenges, and needs (SCN) data within nursing and interprofessional care [15] as well as population health measurement, accelerating the movement toward strength-oriented care and recognizing resilience.

Prior research has led to the development of a consumer-facing app MyStrengths + MyHealth (MSMH) [16] to standardize SCN data capture from a whole-person perspective using SOST [17-19]. Within MSMH and SOST, the integrity and rigor of the structure and concepts of the Omaha System are retained. The Omaha System is a multidisciplinary health terminology [11] that includes three components, the Problem Classification Scheme and related signs/symptoms, the Intervention Scheme, and the Problem Rating Scale for Outcomes. The 42 problem concepts in the Omaha System are organized within four domains (environmental, psychosocial, physiological, and health-related behaviors). These were simplified in SOST as My Living, My Mind and Networks, My Body, and My Self-care (Textbox 1).

**Figure 1:**
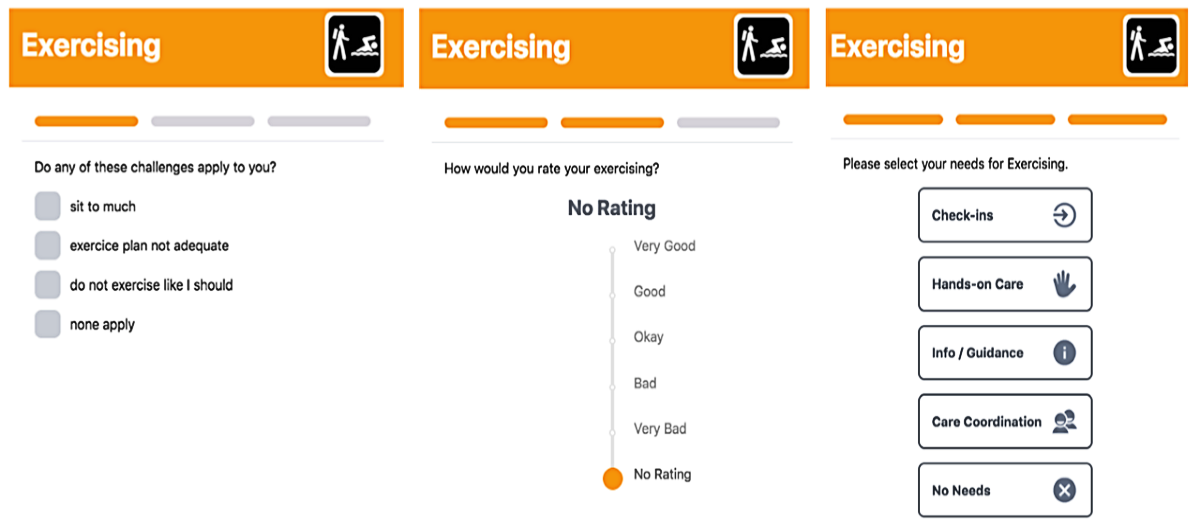
MyStrengths + MyHealth app screenshots with challenges, strengths, and needs for exercising.

The Omaha System Problem Classification Scheme defines the 42 concepts, each of which has 3 to 18 unique taxonomically assigned signs/symptoms/challenges (Figure 1). The Problem Rating Scale for Outcomes enables measurement of strengths across all concepts. A user rates each concept using the Likert-type ordinal scale, where 1 is very bad and 5 is very good (Figure 1). A rating of 4 (minimal challenges) or 5 (no challenges) is defined as a strength. The Intervention Scheme classifies needs (actions) to address all concepts: surveillance (check-ins); treatments and procedures (hands-on care); teaching, guidance, and counseling (info/guidance); and case management (care coordination). In the MSMH app, needs are expressed in four categories: info/guidance (I could use more information about this or some guidance in deciding what to do), hands-on care (hands-on care or help), check-ins (someone to check in with me); and care coordination (help managing my appointments and connections). A screenshot of SCN assessments for the *Exercising* concept is portrayed in Figure 1, and Textbox 2 presents the connections across the SOST domains, concepts, strengths, challenges, and needs.

The MSMH is freely available to researchers, educators, and clinicians, and is licensed through the university [16]. MSMH data are housed in a secure computing network from which license holders may download their complete data. Studies based on the MSMH app have detailed the development and pilot testing [18], analyzed women’s cardiovascular health using the app [20], described whole-person health of older adults [21], and examined local data on whole-person health and opioids in the community [22]. Recent research has analyzed MSMH data for resilience at the community level [7] and examined the feasibility of using MSMH-aided consumer-generated data for knowledge discovery [23]. Researchers have used the Omaha System data to understand whole-person health [15], characterize strengths of older adults with chronic illness [24], and examine relationships between social determinants and health disparities [25].

With the growing interest in whole-person health, emotional well-being, and strengths-based care, there is a need to build a body of evidence to demonstrate the value of informatics tools such as the MSMH app. The objectives of this research were to use visualization techniques and data from attendees at a Midwest state fair to examine SCN overall and by groups with/without *Emotions* challenges and explore the resilience of participants.

**MyStrengths + MyHealth domains and concepts.**

**My Living**

- Income
- Cleaning
- Home
- Safe at home and work

**My Mind and Networks**

- Connecting
- Socializing
- Role change
- Relationships
- Spirituality or faith
- Grief or loss
- Emotions
- Sexuality
- Caretaking
- Neglect
- Abuse
- Growth and development

**My Body**

- Hearing
- Vision
- Speech and language
- Oral health
- Thinking
- Pain
- Consciousness
- Skin
- Moving
- Breathing
- Circulation
- Digesting
- Bowels
- Kidneys or bladder
- Reproductive health
- Pregnancy
- Postpartum
- Infections

**My Self-care**

- Nutrition
- Sleeping
- Exercising
- Personal care
- Substance use
- Family planning
- Health care
- Medications

**Connecting domains, concepts, strengths, challenges, and needs in MyStrengths + MyHealth app.**

**Domains and number of concepts (total number of concepts: 42)**

- My Living (4 concepts)
- My Mind and Networks (12 concepts)
- My Body (18 concepts)
- My Self-care (8 concepts)

**Strengths (42 strengths possible/person)**

- Each concept is rated on a scale of 1-5, with a 4 or 5 rating noted as a strength

**Challenges (335 challenges possible/person)**

- Each concept has 2-18 list of challenges

**Needs (168 needs possible/person)**

- Each concept has 4 needs: check-ins, hands-on care, info/guidance, and care coordination

## Methods

### Ethics Approval

The cross-sectional visualization survey study received approval from the university’s institutional review board (approval number: #STUDY00009465). Researchers collected deidentified data using the SOST and MSMH app.

### Study Setting: Midwestern State Fair

Data collection occurred at a popular Midwest state fair event that is held over 12 days and attracts attendance of more than 2 million annually. This study was conducted in August and September 2021. Participants self-selected to participate in the study. Participation was restricted to adults (18 years or older) who could complete the MSMH app in English. An informed consent was displayed in the app; participants agreed to reuse their anonymous data for research prior to completing the assessment. They completed the MSMH assessment on university-owned iPads. Participants were given a university-branded drawstring backpack (US $1.79 value) as an incentive for participation. At the end of the assessment, a unique code was displayed that participants could use to download a summary of their responses. The link to download the summary along with the participants’ unique code was provided to each participant in a business card format. Appropriate COVID-19 protocols were followed: masks were mandatory, iPads were sanitized after every use, and hand-sanitizing lotions were available in multiple places.

### Study Tool: MSMH App

As displayed in Figure 1, the MSMH app presents SCN for each concept as previously described using the SOST [21,23]. If all concepts are rated, there would be a total of 42 strengths, 335 challenges, and 168 needs. Of these, 37 concepts were chosen for analysis based on the study objectives and the setting/sample, excluding 5 concepts that did not apply to the majority of participants (*Pregnancy*, *Postpartum*, *Family Planning*, *Consciousness*, and *Growth and Development*). The total time to complete the assessment was approximately 15 minutes per user.

### Study Approach and Data Analysis

Data were stored in a secure computing environment hosted by the university. Data were analyzed using visualization techniques in Excel (Microsoft Corporation), and descriptive and inferential statistics were analyzed using SPSS (IBM Corp).

For aim 1, the overall SCN were examined for all participants using descriptive statistics and parallel coordinates visualization techniques [7,26]. Two cohorts were then formed for participants with one or more *Emotions* challenges and those without *Emotions* challenges. SCN were compared using independent sample *t* tests and parallel coordinates visualization techniques. In examining whole-person health and the *Emotions* concept using data visualization techniques, a novel cluster of health-related behaviors domain concepts (*Sleeping*, *Nutrition*, and *Exercising*) were uncovered and were examined in detail. In addition, bubble charts were created to visualize relationships among SCN across the four domains (My Living, My Mind and Network, My Body, and My Self-care).

For aim 2, to examine resilience, a correlational analysis was conducted on the strengths of participants with and without *Emotions* challenge. Co-occurrences of various strengths across the 37 study concepts were analyzed using the Pearson correlation coefficient. The resulting correlation matrix was conditionally formatted in Excel with blue (most correlated), white (midrange), and red (least correlated).

## Results

### Overview

The study participants (N=182) were primarily female (n=123, 67.6%), aged ≥45 years (n=112, 61.5%), White (n=154, 84.6%), and non-Hispanic (n=177, 97.3%). Almost half of the respondents indicated their marital status as married (n=84, 46.2%). The demographics of participants by age, gender, race, ethnicity, and marital status with and without *Emotions* challenges are presented in Table 1. Compared to those without *Emotions* challenges, those with *Emotions* challenges were aged 18-44 years (*P*<.001), more often female (*P*=.04), and not married (*P*=.02). The *Emotions* challenges identified by participants are highlighted in Table 2, with tired (n=69) and hard to manage my stress (n=46) identified as the top two challenges.

**Table 1:** Demographics of participants: overall and by group with and without an emotions challenge.

Sample characteristic		Sample (N=182), n (%)	Without an *Emotions* challenge, n (%)	With an *Emotions* challenge, n (%)	Difference by characteristic and the emotions challenge	
					Chi-square (*df*)	*P* value
**Age (years)**					14.49 (1)	<.001
	18-44	70 (38.5)	18 (9.9)	52 (28.6)		
	45 to ≥65	112 (61.5)	61 (33.5)	51 (28.0)		
**Gender**					4.17 (1)	.04
	Female	123 (67.6)	47 (25.8)	76 (41.8)		
	Male/other	59 (32.4)	32 (17.6)	27 (14.8)		
**Race**					1.71 (1)	.19
	White	154 (84.6)	70 (38.5)	84 (46.2)		
	All other	28 (15.4)	9 (4.9)	19 (10.4)		
**Ethnicity**					0.02 (1)	.88
	Non-Hispanic/ non-Latinx	177 (97.3)	77 (42.3)	100 (54.9)		
	Hispanic/Latinx	5 (2.7)	2 (1.1)	3 (1.6)		
**Marital status**					5.12 (1)	.02
	Married	84 (46.2)	44 (24.2)	40 (22.0)		
	Other marital categories	98 (53.8)	35 (19.2)	63 (34.6)		

**Table 2:** Emotions challenges identified by participants.

Emotions challenges in participants	Participants, n
Tired	69
Hard to manage my stress	46
Hard to concentrate	31
Nothing excites me	27
Mood swings	23
Very sad, hopeless	16
Not interested in taking care of myself	16
Fearful	14
Hard to not repeat things I do	13
Strongly annoyed and acting out	11
Angry	8
Flashbacks	7
Hard to understand real life	6
See or hear things that others cannot	4
I think about killing myself or others	5
Self-harm	5

### Aim 1: Overall Strengths, Challenges, Needs, and With/Without Emotions Challenges

**Figure 2:**
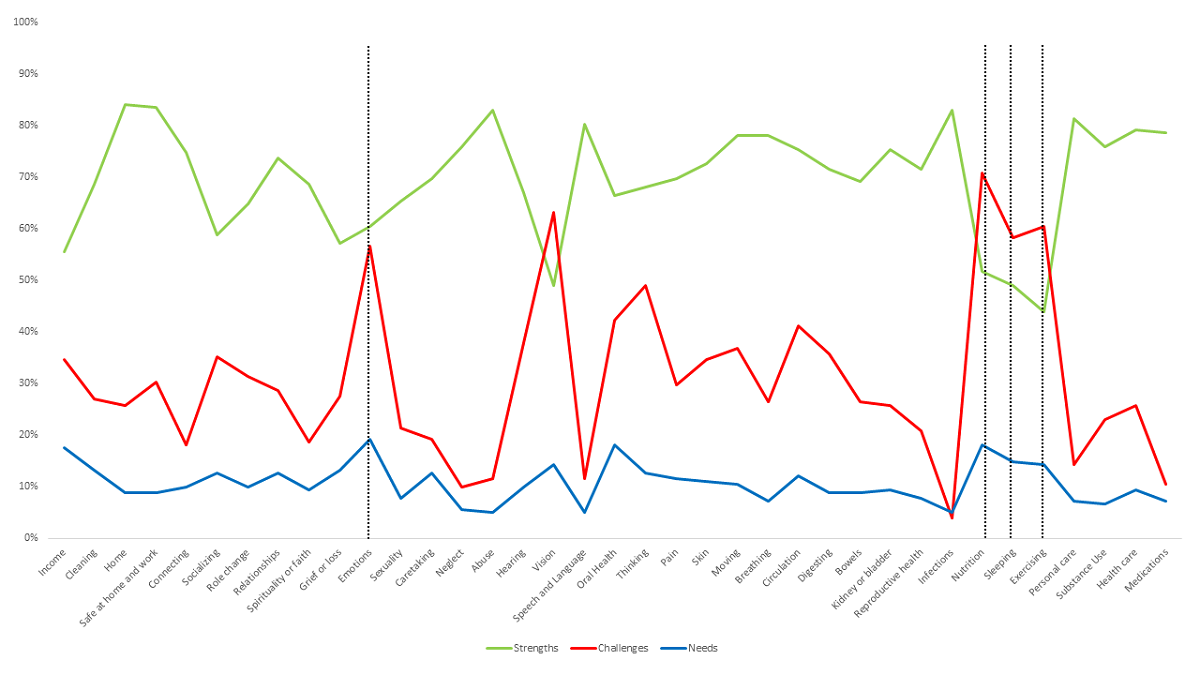
Overall strengths, challenges, and needs.

Figure 2 presents the overall SCN of participants.

#### Strengths

Participants had an average of 28 strengths (mean 28.6, SD 10.5). *Home* (n=152, 83.5%) and *Safe at Home and Work* (n=152, 83.5%) were the two concepts with the most strengths.

#### Challenges

Participants had an average of 12 challenges (mean 12, SD 7.5). Common challenges were *Nutrition* (n=129, 70.9%), followed by *Exercising* (n=109, 60.4%) and *Sleeping* (n=106, 58.2%). Figure 2 displays the challenges (red line) exceeding the strengths (green line) for this cluster of health-related behaviors domain concepts. Over half (n=103, 56.6%) of the participants had one or more challenges in the *Emotions* concept.

#### Needs

**Figure 3:**
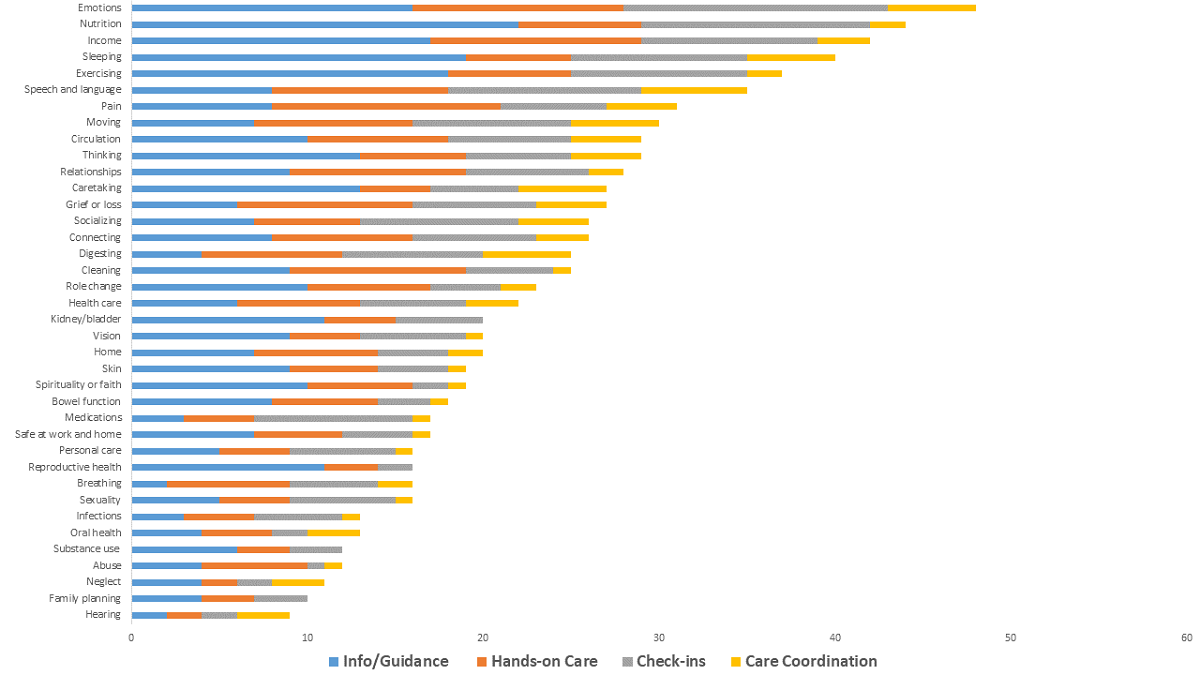
Most frequent needs by concepts.

Participants had an average of 4 needs (mean 4.2, SD 7.5). The *Emotions* concept had the most needs, with info/guidance being the common need for this concept (Figure 3). One out of five (n=35, 19.2%) participants identified a need related to *Emotions*. Overall, the top five needs were in *Emotions*, *Nutrition*, *Income*, *Sleeping*, and *Exercising* (Figure 3).

Overall, participants had more strengths than challenges and needs (Table 3). Compared to those without *Emotions* challenges, those with *Emotions* challenges had fewer strengths, more challenges, and more needs than those without *Emotions* challenges (*P*<.001 for all; Table 3).

**Figure 4:**
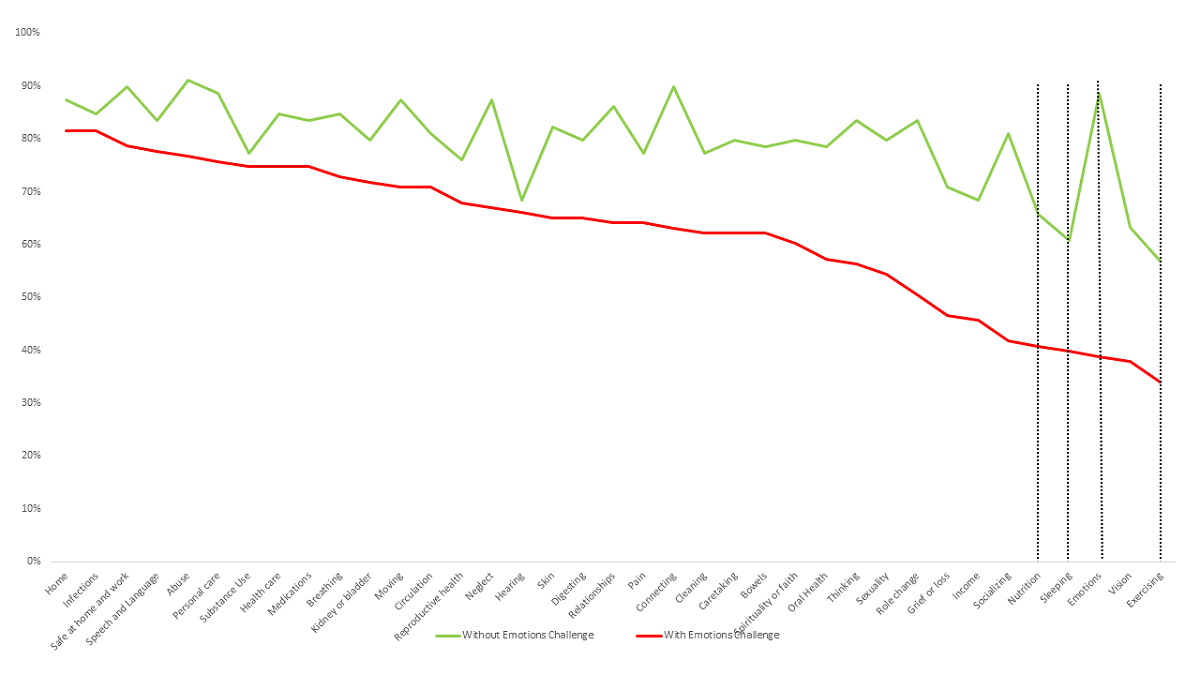
Strength by concept and by group with and without an Emotions challenge.

The analysis of strengths across concepts and by group with/without *Emotions* challenge showed that the group without *emotions* challenge (indicated in green in Figure 4) had more strengths across all concepts. Compared to those without *Emotions* challenges, those with *Emotions* challenges had fewer strengths for *Emotions* (*P*<.001) and for the cluster of health-related behaviors domain concepts: *Sleeping* (*P*=.002), *Nutrition* (*P*<.001), and *Exercising* (*P*<.001).

**Figure 5:**
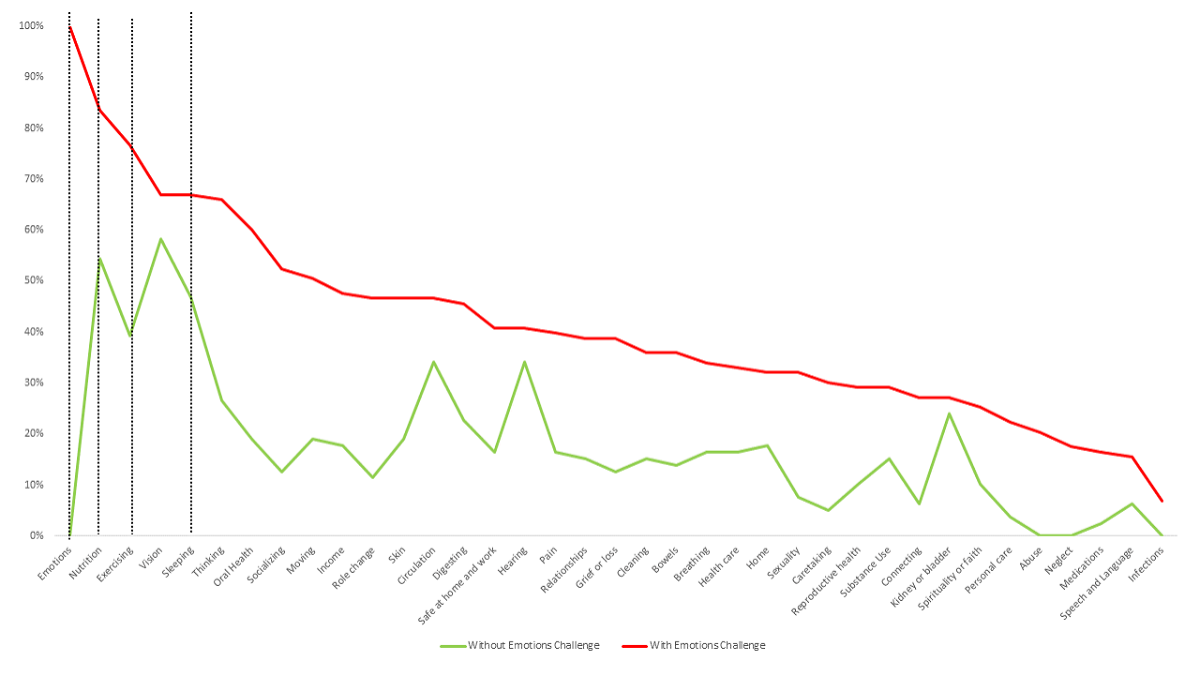
Challenges by concept and by group with and without Emotions challenges.

Figure 5 displays the analysis of challenges across the two groups, with higher challenges across all concepts for those in the *Emotions* challenge group (indicated by the red line). The group with the *Emotions* challenge had more challenges. Compared to those without *Emotions* challenges, those with *Emotions* challenges had more challenges on the cluster of health-related behaviors domain concepts: *Sleeping* (*P*=.003), *Nutrition* (*P*<.001), and *Exercising* (*P*<.001).

**Figure 6:**
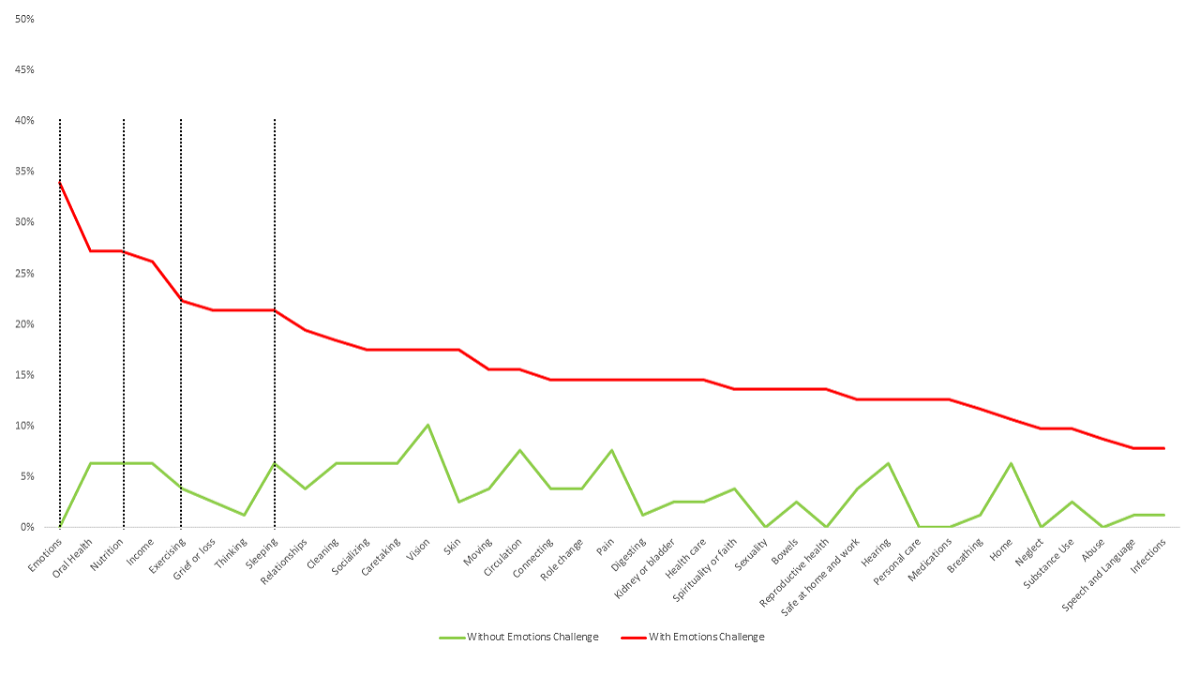
Needs by concept and by group with and without Emotions challenges.

The analysis of needs showed more needs for all concepts in the group with *Emotions* challenges (Figure 6). Compared to those without *Emotions* challenges, those with *Emotions* challenges had more needs for *Emotions* (*P*<.001), and health-related behaviors domain concepts: *Sleeping* (*P*=.002), *Nutrition* (*P*<.001), and *Exercising* (*P*<.001).

**Figure 7:**
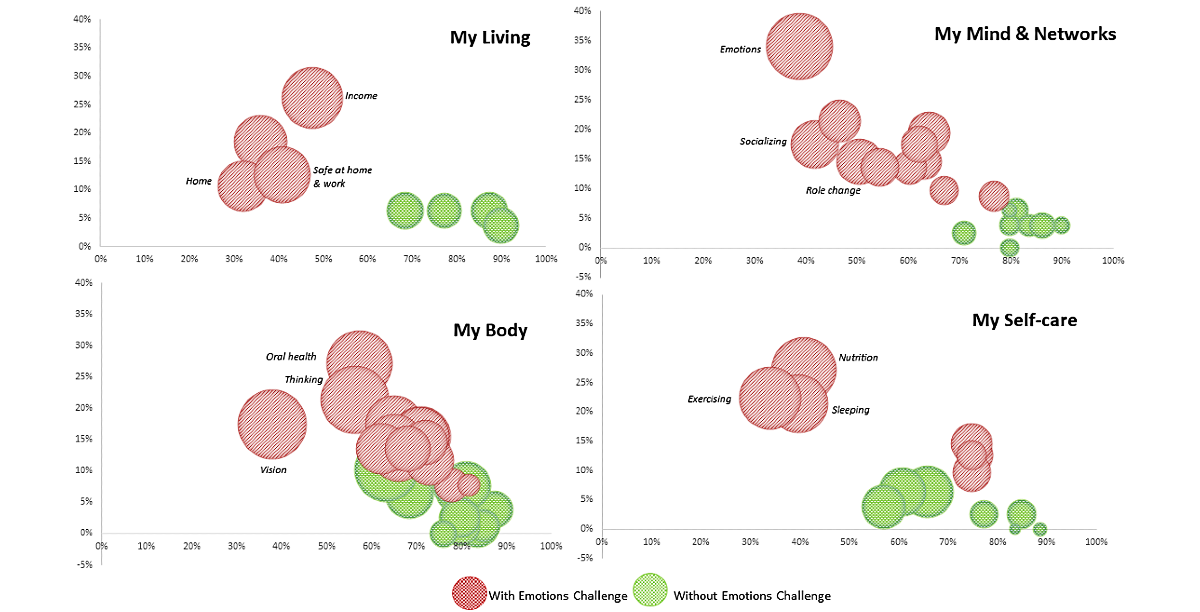
Strengths, challenges, and needs by domain, with and without Emotions challenges.

Additional visual analysis was conducted across the four MSMH domains to understand whole-person health by domain and concept using bubble charts (Figure 7) in which larger bubble size indicated more challenges; being on the left end of the x-axis showed fewer strengths and on the higher end of the y-axis showed greater needs. This is much more pronounced for the group with *Emotions* challenges (red bubbles) as shown in Figure 7. The combined patterns of location on the two axes and the bubble size show pronounced difference by problem: bubble size and location were notably larger, higher, and more left for *Income* in the My Living domain; *Emotions* and *Socializing* in the My Mind and Networks domain; *vision* and *thinking* in My Body; and *Sleeping*, *Nutrition*, and *Exercising* in My Self-care.

**Table 3:** Strengths, challenges, and needs: overall and by group with and without an emotions challenge.

Variable	Overall (N=182), mean (SD)	Without an *Emotions* challenge (n=79), mean (SD)	With an *Emotions* challenge (n=103), mean (SD)	*P* value
Strengths	28.6 (10.5)	32 (9.6)	26 (10.4)	<.001
Challenges	12 (7.5)	6.7 (3.9)	16.2 (7.0)	<.001
Needs	4.2 (7.5)	1.4 (2.4)	6.3 (9.2)	<.001

### Aim 2: Characterize Resilience for Groups With and Without an Emotions Challenge

**Figure 8:**
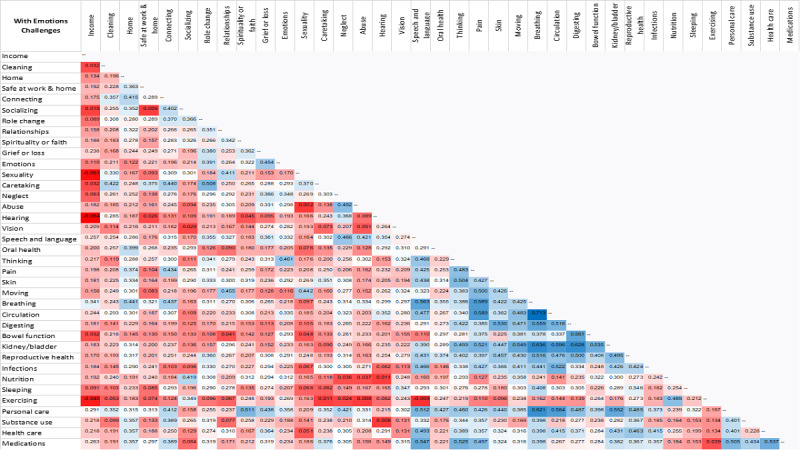
Strength correlations with Emotions challenges.

**Figure 9:**
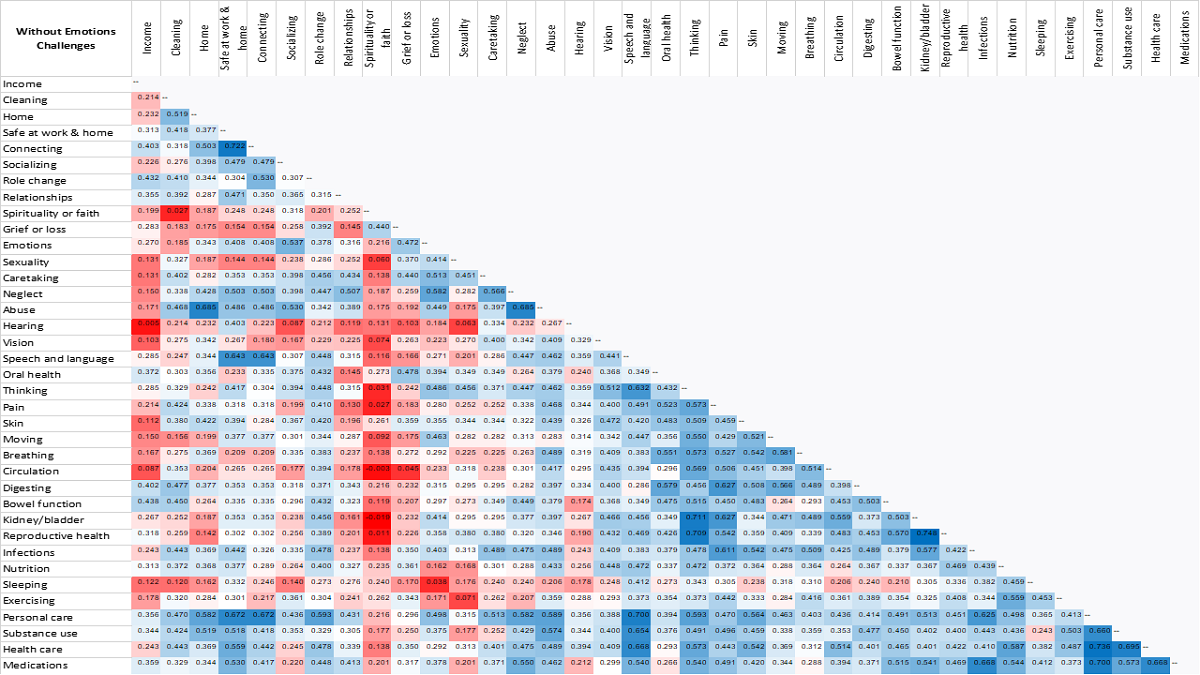
Strength correlations without Emotions challenges.

The correlational analysis to identify associations in strengths for participants with and without *Emotions* challenges are presented in Figures 8 and 9, respectively. A higher correlation indicates co-occurrence of strengths [7,27]. A total of 666 boxes are displayed for strength correlations with *Emotions* challenges and likewise for without *Emotions* challenges. A red box is an indicator of less correlation and a blue box is an indicator of high correlation, with the white box being in between. For those with *Emotions* challenges, 22.5% (n=150) are blue boxes out of the total of 666 boxes, and for those without *Emotions* challenges, 51.7% (n=344) are blue boxes. As shown in Figures 8 and 9, the correlations among concepts are less strong for those with *Emotions* challenges (more red boxes) and stronger for those without *Emotions* challenges (more blue boxes indicating more and greater positive correlations). The correlation matrix for the group without an *Emotions* challenge depicts strengths that extend across concepts/domains as indicated by the blue boxes that span horizontally and vertically.

## Discussion

### Findings and Implications

In this descriptive correlational study, SCN data from participants in a Midwest state fair over August-September 2021 were examined using visualization techniques. Overall, participants had more strengths than challenges and more challenges than needs. The data visualization techniques used to examine whole-person health and the *Emotions* concept revealed a novel cluster of health-related behaviors (My Self-care) domain concepts (*Sleeping*, *Nutrition*, and *Exercising*) with fewer strengths and more challenges and needs. This aligns with the association between sleep, physical activity, and diet during COVID-19 [28-30]. Furthermore, differences in SCN for *Sleeping* in groups with/without an *Emotions* challenge aligns with prior research on the impact of sleeping on overall mental health [31,32]. It is notable that more than half of the participants had challenges in the *Emotions* concept. That those with *Emotions* challenges had fewer strengths and more challenges and needs across all concepts underscores the importance of a whole-person health perspective. Further research is needed to understand nuances related to SCN across and among these concepts in respect to whole-person health.

The finding that more than half of the participants had challenges in the *Emotions* concept underscores the impact and emotional toll of the pandemic. This aligns with the evidence that emotional issues such as depression, anxiety, and suicidal thoughts have increased since 2020 [2]; for example, the number of US adults who reported symptoms of anxiety or depression in January 2021 increased 4 times compared to June 2019 [2]. The How Right Now communications campaign by the Centers for Disease Control and Prevention [2] aims to promote and strengthen the emotional well-being and resilience of people affected by stress, grief, and loss during the COVID-19 pandemic. Data on whole-person health such as those offered by this study has the potential to provide a quantitative data perspective to add to the qualitative lived experiences/narratives being shared as part of this initiative.

The finding that those with *Emotions* challenges also had many strengths is promising; and it is important to identify these as a tool to help understand and potentially bolster resilience. These findings align with prior strength-oriented studies [14,15,33-35]. Strengths can be used as tools to counter challenges, as an individual who has strength in *Socializing* or *Spirituality or Faith* is more likely to use social support systems to mitigate challenges related to concepts such as *Emotions*, *Relationships*, or *Grief or loss* and use these as their mechanisms to preserve and heal and, hence, build resilience [36,37].

The finding that those with *Emotions* challenges have fewer strengths and more challenges and needs across all four domains compared to those without *Emotions* challenges aligns with the literature regarding the impact of mental health on all of health [38,39]. Results align with prior studies that health and disease are a dynamic interconnected state with a ripple effect on other aspects of health [3,40]. Tools such as SOST and MSMH can provide a whole-person assessment and identify areas of strengths to leverage and help boost resilience. The SOST terminology within the MSMH app enabled comparisons across communities and clinical data [7,22], and has been used to analyze community-level resilience [7]. Given the potential for assessment at the individual and community level, powerful tools such as SOST and the MSMH app should be adopted within EHRs and personal health records to generate meaningful data for population health management.

This aligns with the current National Institutes of Health Bridge2AI Initiative that aims to build and leverage robust multidimensional data sets [41]. By tapping into increasing computing power along with machine learning, artificial intelligence, and transformative analytic techniques, these data sets can be used to draw insights on factors that facilitate whole-person health and resilience. Apps such as MSMH leverage the power of terminology standards and health informatics, and provide options for addressing the recommendations of the National Academies of Sciences, Engineering, and Medicine on design and use of health information technology for whole-person health [42].

### Strengths and Limitations

This research demonstrates the power of health informatics, standardized data, and technology to assess, visualize, and test individual- and community-level data. It demonstrates a data-driven methodology for additional studies to build evidence on whole-person health and resilience. Furthermore, such research offers a starting point for initiating conversations about whole-person health with individuals and communities regarding their strengths, challenges, and needs, beginning a shift from a deficit model of health toward whole-person health.

Some limitations were noted. Recruitment challenges due to COVID-19 were considerable, given limited attendance at the state fair and the fact that participants needed to participate indoors and wear a mask inside the research building. The sample may have been biased by these pandemic conditions, as fair attendees in 2021 may be unique in some way that is not known. Lastly, the survey took 15 to 20 minutes to complete, which was a deterrent to some potential participants.

### Conclusions

This study examined standardized whole-person data using an app-aided and data-driven approach, quantifying SCN of individuals across all of health (environmental, psychosocial, physiological, and health-related behaviors domains). Examining SCN data for groups with and without *Emotions* challenges revealed patterns in overall health and for important health-related behaviors concepts. This study lays a foundation for numerous research opportunities, such as metric development to measure resilience and the use of SOST and MSMH in clinical care settings to reframe health care encounters in a whole-person perspective.

## Abbreviations

EHR: electronic health record
MSMH: MyStrengths + MyHealth
SCN: strengths, challenges, and needs
SNOMED CT: Systemized Nomenclature of Medicine–Clinical Terms
SOST: Simplified Omaha System Terms
